# School-Based Countrywide Seroprevalence Survey Reveals Spatial Heterogeneity in Malaria Transmission in the Gambia

**DOI:** 10.1371/journal.pone.0110926

**Published:** 2014-10-22

**Authors:** Joseph Okebe, Muna Affara, Simon Correa, Abdul Khalie Muhammad, Davis Nwakanma, Chris Drakeley, Umberto D’Alessandro

**Affiliations:** 1 Disease Control & Elimination Theme, Medical Research Council Unit, Fajara, The Gambia; 2 Department of Immunology and Infection, London school of Hygiene and Tropical Medicine, London, United Kingdom; 3 Department of Disease Control, Faculty of infectious and Tropical Diseases, London school of Hygiene and Tropical Medicine, London, United Kingdom; 4 Department of Public health, Institute of Tropical Medicine, Antwerp, Belgium; Brighton and Sussex Medical School, United Kingdom

## Abstract

**Background:**

As the geographical distribution of malaria transmission becomes progressively clustered, identifying residual pockets of transmission is important for research and for targeting interventions. Malarial antibody-based surveillance is increasingly recognised as a valuable complement to classic methods for the detection of infection foci especially at low transmission levels. The study presents serological evidence for transmission heterogeneity among school children in The Gambia measured during the dry, non-transmission season.

**Methods:**

Healthy primary school children were randomly selected from 30 schools across the country and screened for malaria infection (microscopy) and antimalarial antibodies (MSP1_19_). Antibody distribution was modelled using 2-component finite mixture model with cut-off for positivity from pooled sera set at 2-standard deviation from the mean of the first component. Factors associated with a positive serological status were identified in a univariate model and then combined in a multilevel mixed-effects logistic regression model, simultaneously adjusting for variations between individuals and school.

**Results:**

A total of 4140 children, 1897 (46%) boys, were enrolled with mean age of 10.2 years (SD 2.6, range 4–20 years). Microscopy results available for 3640 (87.9%) children showed that 1.9% (69) were positive for *Plasmodium falciparum* infections, most of them (97.1%, 67/69) asymptomatic. The overall seroprevalence was 12.7% (527/4140) with values for the schools ranging from 0.6% to 43.8%. Age (OR 1.12, 95% CI 1.07–1.16,) and parasite carriage (OR 3.36, 95% CI 1.95–5.79) were strongly associated with seropositivity.

**Conclusion:**

Serological responses to malaria parasites could identify individuals who were or had been infected, and clusters of residual transmission. Field-adapted antibody tests able to guide mass screening and treatment campaigns would be extremely useful.

## Introduction

In the past decade, there has been a significant but uneven reduction in malaria indicators [1], [2] following the scale up of effective interventions, partly attributed to underlying heterogeneities in malaria transmission [3]. Understanding the dynamics of transmission in places with such heterogeneity could help to explain why similar interventions have differential impact and support targeted control efforts. An important first step is to determine the tools and methods that can efficiently detect these variations in malaria transmission [4].

Classically, malaria endemicity and transmission are described by the parasite prevalence and the entomological inoculation rate (EIR) respectively, and these methods remain useful in many settings. Nevertheless, in low transmission settings, the paucity of infected mosquitoes, the need to collect and analyse large number of samples coupled with declining sensitivities of microscopy, RDTs and EIRs reduce the efficiency of these methods [5]. Estimating the prevalence of antimalarial antibody (seroprevalence) is increasingly recognised as a valuable complement to classic methods for defining transmission intensity [6], [7], determining heterogeneity in outcomes of malaria interventions [8] and for malaria surveillance [9].

Where malaria transmission is stable, children less than 5 years old bear the greatest burden of malaria so the intensity of malaria transmission and the impact of interventions are often established in this age group. However, with decreasing transmission, older children may become increasingly at risk of malaria [1], [10]. Therefore, determining the seroprevalence in this age group may be extremely useful for estimating short-term changes in the burden of infection over a broad location [11], [12]. In this context, school-based serological surveys may be an effective and operationally attractive alternative to population-based surveys for identifying areas with varying transmission [13]. A pilot survey of 32 schools in the Upper River Region; one of the six administrative regions of The Gambia, showed that transmission in the area was quite heterogeneous and identified potential foci of transmission [14].

As part of a number of ongoing studies on the dynamics of transmission in low endemic settings, the methods previously tested in the pilot study were applied in a nationwide schools survey to describe and document the variation in malaria transmission across the whole country. This paper reports on the results and discusses the use of seroprevalence data to describe trends in transmission.

## Methods

In May 2012, at the end of the dry season and before the onset of the rains, a cross-sectional malaria seroprevalence survey was carried out among primary school pupils across The Gambia. The country has a population of about 1.8 M people with 39% less than 15 years old [15].

**Figure 1:**
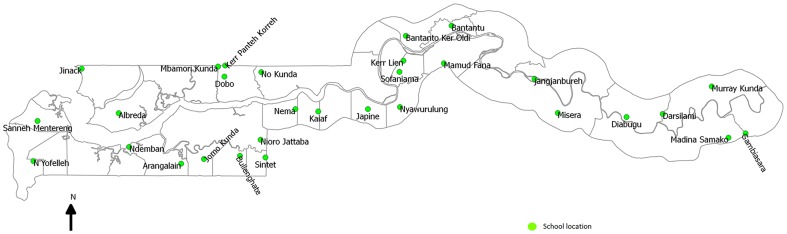
Geographical locations of primary schools sampled in the survey. Circles represent the GPS location of the school.

Summary data on school attendance by district and region was obtained from the Ministry of Education. There are 411 primary schools distributed across 37 educational districts in six administrative regions [15]; seven of these districts (one region) along the coast were excluded from the survey because the distribution of pupils’ residence overlapped between the location of schools such that the latter could not be used as a distinct marker of geographical catchment. Based on data, the median number of children in each school was 229 (range 150–1126). One primary school per remaining district was then randomly selected and in each school, 150 children were randomly selected from the school register using a set of random numbered cards (Figure 1). An advance field team prepared 150 cards bearing “yes” on them and identical blank cards up to the number of children in each school. Each child was asked to select a card from the pile and any child who drew a “yes” card, was selected to participate.

A copy of the information sheet describing the study together with an invitation to meet the study team on a pre-defined date at the school premises were distributed to the caregivers of the selected children who were asked to take them home. On the scheduled day, members of the research team met with the caregivers, explained the study, answered questions and asked for a written informed consent for their children to participate to the survey.

Consenting caregivers were asked about bed net availability and use at home, and indoor residual spraying (IRS) of their homes in the preceding year. Enrolled children had their body temperature checked and a blood sample collected by fingerpick for slide microscopy, haemoglobin (Hb) estimation with a portable device (HemoCue AB, Ängelhom, Sweden) and on to a filter paper for subsequent antimalarial antibody measurement. Febrile children (temperature >37.5°C) were screened for malaria using a *Paracheck-Pf* rapid test kit (Orchid Biomedical Systems, Goa, India) and an antimalarial give if positive, where negative; the study nurse gave treatment based on the most likely alternative diagnosis.

### Laboratory procedures

Blood slides were stained with 10% Giemsa for 10 minutes and read independently by two microscopists. The presence of *P. falciparum* malaria parasites was determined by reading 100 high power fields under oil immersion. Where a log_10_ difference in parasite density between the two reads was noted, a third read by a senior microscopist was used to resolve the result.

Antibodies against merozoite surface protein-1_19_ (MSP1_19_) were determined by indirect ELISA with samples eluted from collected filter papers using previously published methods [14].

### Sample size and statistical analysis

A previous dry season survey in the eastern part of the country showed the prevalence of MSP1_19_ antibodies to be about 20% [10]. Considering that the prevalence was likely to vary in locations across the country, the sample size was calculated to detect this prevalence with varying levels of precision ranging between 1% (95% CI 0.2–5%) and 20% (14–27%), adjusting for clustering by school.

Collected field data were double entered into a database on OpenClinica (Akaza Research, USA). After cleaning, the data was analysed using STATA version 12.1 (College Station, Texas). Summary characteristics were presented for continuous (means and standard deviations) and categorical (proportions) variables for each school.

Antimalarial antibody distribution was modelled by fitting pooled positive sera using a two-component finite mixture model and the cut off for positivity set at two standard deviations away from the mean of the first component. This derived cut-off titre was applied across all children to determine proportion of seropositive children in the study population and in each school. The prevalence of parasitaemia (microscopy), seropositivity and anaemia (Hb <11 g/dl) across schools and the correlation between malaria infection and seroprevalence were determined.

Factors associated with a positive serological status were identified in a univariate model and then combined in a multilevel mixed-effects logistic regression model, which simultaneously adjusts for variations between individual children and across schools. The goodness of fit was measured by calculating the Pearson residual and by visually assessing the agreement between these parameters using a Bland-Altman plot.

### Ethics statement

The Gambia Government/MRC Joint Ethics Committee approved the study (SCC 1318) and the Directorate of Basic and Secondary Education at the Ministry of Basic and Secondary Education granted access to the schools. A written informed consent to participate was obtained from caregivers on behalf of all children enrolled in the study.

## Results

A total of 4140 children were enrolled, 1897 (46%) were boys. The ages of the children ranged between 4 and 20 (mean 10.2, SD 2.6) years and were similar between schools. The mean bednet ownership was 87% (range 42%–99%) and the large majority (85.6%, 3545/4140) of homes had reportedly been sprayed with an insecticide within the previous year (Table 1).

**Table 1:** Baseline characteristics and prevalence of malaria infection, anaemia and malaria antibodies by school.

School	No. enrolled	Male n(%)	Bednet ownership n(%)	IRS* n(%)	Anaemic§ n(%)	Slide positive# n(%)	Mean Hb (SD)	Sero-positive n(%)
Jomo Kunda	104	49 (47)	100 (96.2)	96 (93.2)	1 (1.0)	2 (2.0)	13.4 (0.9)	2 (1.9)
Bullenghate	163	86 (53)	144 (88.3)	160 (98.8)	4 (2.5)	0 (0.0)	13.0 (1.1)	4 (2.5)
Sintet	137	48 (35)	127 (92.7)	134 (98.5)	4 (2.9)	0 (0.0)	13.0 (0.9)	5 (3.6)
Sanneh Mentereng	39	20 (51)	30 (76.9)	16 (41.0)	0 (0.0)	1 (4.5)	13.2 (1.0)	2 (5.1)
Arangalain	135	66 (49)	100 (74.1)	94 (71.2)	4 (3.0)	2 (1.5)	13.3 (1.0)	7 (5.2)
N'Yofelleh	74	47 (64)	49 (66.2)	50 (67.6)	1 (1.4)	1 (1.4)	13.1 (0.9)	7 (9.5)
Ndemban	152	66 (43)	143 (94.1)	120 (79.5)	5 (3.3)	3 (3.2)	13.0 (1.2)	18 (11.8)
Kerr Pateh Korreh	154	79 (53)	134 (87.0)	138 (93.9)	12 7.8)	0 (0.0)	12.7 (1.5)	1 (0.6)
No Kunda	156	86 (55)	127 (81.4)	151 (97.4)	2 (1.3)	0 (0.0)	13.1 (1.0)	5 (3.2)
Jinack	133	63 (47)	112 (84.2)	116 (87.2)	4 (3.0)	0 (0.0)	13.0 (1.1)	5 (3.8)
Mbamori Kunda	154	74 (49)	147 (95.5)	151 (98.7)	10 (6.5)	2 (1.3)	12.5 (1.3)	11 (7.1)
Darsilami	142	80 (56)	137 (96.5)	138 (99.3)	16 (11.3)	0 (0.0)	12.4 (1.4)	10 (7.0)
Albreda	128	67 (52)	120 (93.8)	122 (95.3)	4 (3.1)	1 (0.9)	12.6 (1.0)	10 (7.8)
Nioro Jattaba	150	73 (49)	142 (94.7)	150 (100.0)	16 (10.7)	2 (2.1)	12.2 (1.5)	19 (12.7)
Misera	144	45 (31)	109 (75.7)	144 (100.0)	16 (11.1)	0 (0.0)	12.3 (1.3)	16 (11.1)
Kaiaf	149	76 (51)	137 (91.9)	149 (100.0)	7 (4.7)	1 (0.7)	12.3 (1.0)	23 (15.4)
Nema	150	87 (58)	144 (96.0)	150 (100.0)	9 (6.0)	0 (0.0)	12.6 (1.1)	24 (16.0)
Japine	147	54 (37)	141 (95.9)	147 (100.0)	13 (8.8)	2 (1.4)	12.2 (1.1)	30 (20.4)
Nyawurulung	137	63 (46)	106 (77.4)	137 (100.0)	15 (10.9)	3 (2.3)	12.0 (1.3)	60 (43.8)
Mamud Fana	147	56 (38)	146 (99.3)	143 (97.3)	24 (16.3)	1 (0.7)	12.1 (1.3)	6 (4.1)
Kerr Lien	146	62 (42)	130 (89.0)	143 (97.9)	26 (17.8)	0 (0.0)	12.1 (1.4)	4 (2.7)
Jangjanbureh	148	60 (41)	147 (99.3)	137 (93.2)	21 (14.2)	0 (0.0)	12.2 (1.2)	15 (10.1)
Dobo	135	49 (36)	127 (94.1)	124 (91.9)	27 (20.0)	0 (0.0)	12.1 (1.4)	15 (11.1)
Bantanto Ker Oldi	151	69 (46)	148 (98.0)	145	8 (5.3)	2 (1.4)	12.6 (1.3)	20 (13.2)
Bantantu	142	51 (36)	138 (97.2)	138	12 (8.5)	0 (0.0)	12.4 (1.3)	22 (15.5)
Sofaniama	158	60 (38)	155 (98.1)	158	10 (6.3)	2 (1.6)	12.5 (1.2)	33 (20.9)
Diabugu	150	71 (48)	93 (62.0)	16	17 (11.3)	0 (0.0)	12.4 (1.2)	8 (5.3)
Murray Kunda	125	67 (54)	119 (95.2)	124	17 (13.6)	11 (13.9)	11.9 (1.2)	43 (34.4)
Madina Samako	150	68 (45)	118 (78.7)	54	27 (18.0)	21 (15.0)	12.0 (1.4)	52 (34.7)
Gambisara Lamoi	140	55 (40)	58 (41.4)	0	16 (11.4)	12 (9.1)	12.1 (1.3)	50 (35.7)

IRS*: Indoor residual spray (data from 4117 respondents) Hb Haemoglobin.

§ Haemoglobin <11 g/dl SD Standard deviation.

# Data available for 3640 children.

The mean Hb was 12.5 (SD 1.3) g/dl and 8.4% (348/4140) children had an Hb <11 g/dl; the odds of anaemia were not significantly lower in boys compared to girls (OR 0.88, 95% CI 0.71–1.09). A history of fever in the preceding 24 hours was reported in 4.9% (203/4140) children of whom 4.4% (9/203) had an axillary temperature ≥37.5°C at the time of the survey. These were all negative for malaria using the rapid malaria test kit.

Microscopy results were available for 87.9% (3640/4140) children, 1.9% (69/3640) were positive and all were *Plasmodium falciparum* monoinfections. Most (97.1%, 67/69) of those infected were asymptomatic, i.e. without any history of fever. The odds of infection did not vary with age (OR 0.99, 95% CI 0.90–1.08).

**Figure 2:**
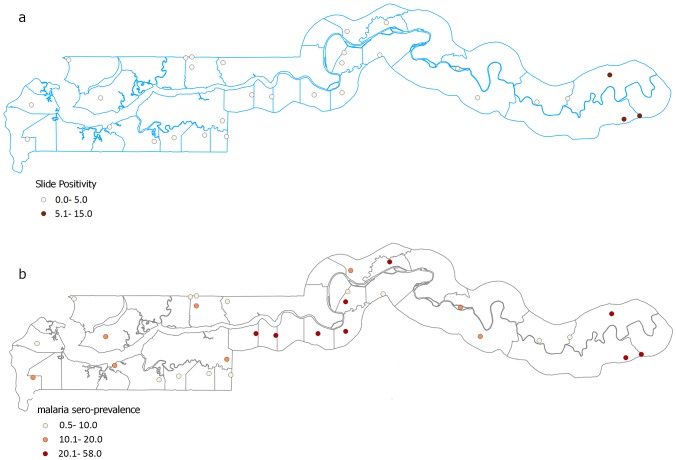
Slide positivity and seroprevalence rates for sampled schools. Circles represent schools location and colour graded according to the inset showing the prevalence of individuals with a positive blood slide for P. falciparum (A) and positive for antibodies to MSP1_19_ (B).

The prevalence of malaria infection, measured by microscopy, was spatially variable with the highest prevalence seen in schools in the eastern part of the country. In 27 of the 30 schools, malaria prevalence was less than 5% (in 13 of these schools, the prevalence was 0%) while in the three remaining schools, the prevalence ranged between 9% and 15% (Figure 2a). Infected children had lower haemoglobin levels compared to the uninfected (OR 0.78, 95% CI 0.67–0.90).

**Figure 3:**
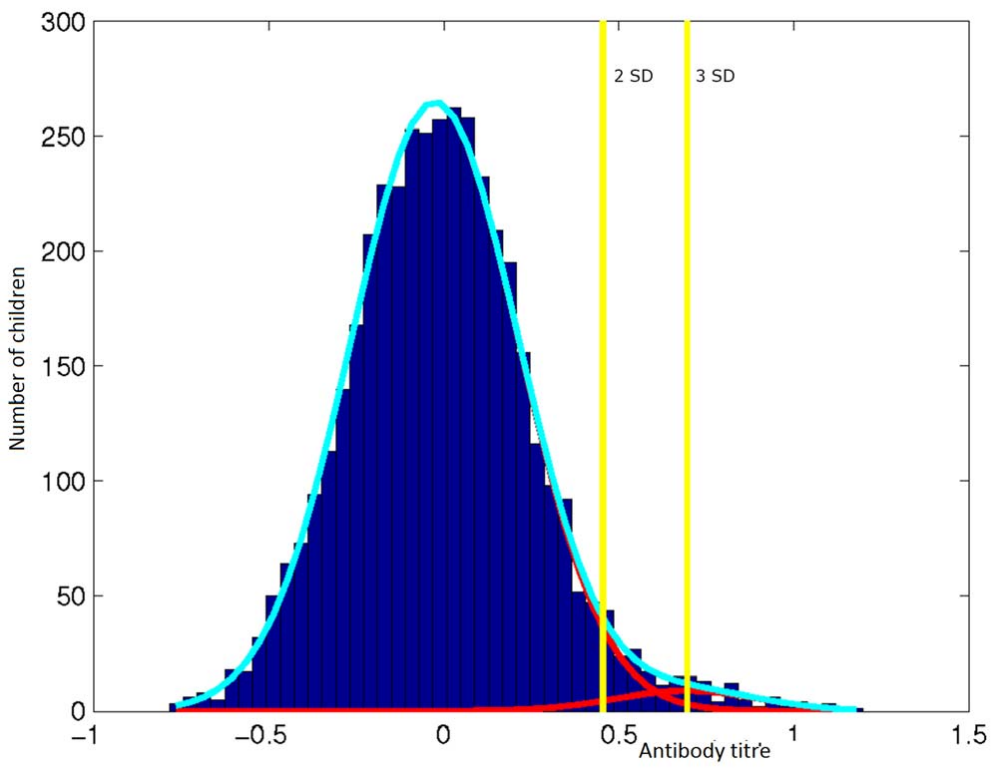
Two-component model distribution of antibody titres (yellow bars: 2 and 3 SD).

From the model, a titre value of 1.884, 2SD from the mean of the first (larger) component (Figure 3) was set as the cut-off defining seropositivity in the population. Based on this value, 12.7% (527/4140) of children were considered positive for MSP1_19_ antibodies and seroprevalence at the schools ranged between 0.6% and 43.8% (Figure 2b). There was a weak correlation between serology and microscopy results (Spearman’s rho =  0.15, P <0.001). The odds of being seropositive increased by 6% with each year of age (OR 1.06, 95% CI 1.03–1.10). The odds of being seropositive was analysed in those at the extremes of age range; <4 (11/4140, 0.3%) years and ≥15 (299/4140, 7.2%) years compared to those 5–14 years. Children less than four years (OR 0.43, 95% CI 0.05–3.50) and older than 15 year (0.61 95% CI 0.39–0.96) had significantly lower odds of being seropositive compared to those aged 5- 15 years. In the regression model, the inclusion of age as these categories did not significantly improve the final model so was not used.

Parasite carriage (a positive slide result) was associated with an 8-fold increase in the odds of being seropositive (OR 7.50, 95% CI 4.63–12.15, Table 2). Children living in homes that reported indoor residual spraying in the previous year were associated with reduced odds of being seropositive (0.65, 95% CI 0.521–0.82).

**Table 2:** Crude and adjusted estimates for the odds of being positive for antimalarial antibodies.

Variables	Crude OR (95% CI)	P value	Adjusted* OR (95% CI)	P value
Age	1.06 (1.03–1.10)	0.001	1.12 (1.07–1.16)	<0.001
Anaemia	0.63 (0.47–0.82)	0.002	0.84 (0.59–1.18)	0.317
Bednet ownership	0.84 (0.65–1.10)	0.212	1.19 (0.85–1.67)	0.319
Indoor residual spray	0.65 (0.51–0.82)	<0.001	0.99 (0.62–1.58)	0.962
Parasitaemia	7.50 (4.63–12.15)	<0.001	3.36 (1.95–5.79)	<0.001

*Adjusted odds ratios are derived from a logistic regression model including all variables.

From the mixed-effects logistic model, age (as a continuous variable), anaemia, bednet ownership, IRS and parasite carriage (positive blood slide), only age (OR 1.12, 95% CI 1.07–1.16) and parasite carriage (OR 3.36, 95% CI 1.95–5.79) remained strongly associated with seropositivity (Table 2).

## Discussion

In settings with seasonal malaria transmission, parasite carriage is extremely low during the dry season and confined to particular locations and individuals [13], and represent residual foci of infection. Typically, the rainy season ends in the month of November although malaria transmission could continue up to February in areas where stagnant pools of water persist. This survey was conducted in the month of May, the end of the dry season and a period when transmission is likely to be at its nadir.

Therefore dry season malaria surveys could provide insights on the duration of parasite carriage as most (if not all) these infections were probably acquired during the previous transmission season, and on where transmission is likely start after the onset of the rains.

Some studies suggest that the proportions of infected and clinically ill persons are higher in these foci than in the surrounding population [6], [16]. However, few studies have showed that tracking reported clinical cases during the dry season could consistently identify these foci [17]. One of the main limitations has been the sensitivity of the existing detection tools.

This is probably the most extensive description of the burden of malaria infection in The Gambia at the end of the dry season when malaria transmission has practically ceased and supports the evidence for heterogeneity and declining transmission across the country [10], [14], [18]. Asymptomatic malaria infections are common, persistent but yet difficult to measure [19]. From the microscopy results, the prevalence of infection was zero in several schools and not very high in all other schools. The schools with distinctly notable prevalence are located towards the eastern border of the country and the values reported are similar to those from earlier surveys in the area [10], [14] and suggest that transmission in the eastern part, compared to the rest of the country, may be relatively unchanged or is declining at a much slower rate.

Such low numbers of infected individuals at the end of the dry season could be targeted to reduce the parasite reservoirs and thus transmission. Nevertheless, there are probably sub-patent infections in these areas, undetected by microscopy, which could still restart transmission [20].

Parasite density in asymptomatic individuals may be influenced by acquired immunity and seasonal variations in transmission, and these could affect the sensitivity of screening tools. Both parasitological (microscopy) and serology data show countrywide variation in transmission but with weak correlation between the two measures. Seropositivity estimates have also been seen to be weakly correlated with estimates by PCR although detection rates are higher in the latter compared to microscopy [14]. This is probable if the very low residual parasite densities (<300 parasites/μl) carried through the dry season is not sufficient to mount an immune response above what would be considered as background for the age and degree of transmission. Also, the results might be more in agreement where more immunogenic malaria antigens rather MSP alone are used. Overall, there was a strong association between parasite and serological prevalence as seropositive children had a 3-fold increase in the odds of parasitaemia detectable by microscopy.

Malaria risk can vary geographically and by both individual and community variables, sometimes with conflicting effects [21]. In contrast to microscopy, serological surveys represent the epidemiological memory of transmission, describing the local short-to-medium term changes that occurred in the study area [12], [22]. The prevalence of antimalarial antibodies differed considerably between schools, which suggest that the varying pattern of transmission may have been persistent for some time. Such islands of intense transmission surrounded by low transmission areas were also observed in a survey of villages surrounding the schools, conducted in the peak of transmission (November 2012), which showed similar trends in terms of geographic location. The mean prevalence of infection, by molecular methods, was 4.3% in the western and 33.7% in the eastern part of the country (manuscript in preparation).

It is likely that transmission will become more heterogeneous as it declines so interventions need to be matched to persons and locations where they are likely to have the maximum effect, [13]. The utility of entomological inoculation rate (EIR) to measure transmission is reduced with declining transmission due to the difficulty of measuring it reliably because of low mosquito densities and low prevalence of infection among them [23], [24]. In such settings, serological estimates could be a suitable alternative as they correlate well with EIRs [11] without the need of carrying out labor-intensive entomological surveys. Moreover, serological surveys can cover a much larger geographical area than entomological surveys, and this is an important factor when considering the heterogeneity of malaria prevalence and transmission.

This data suggests that serological tests could identify areas of persistent malaria transmission even where parasite-infected individuals are undetected by routine tests. Potentially, they could be used either for surveillance in a (pre-) elimination status or as a prelude to interventions, e.g. mass treatment, aiming at to the elimination of the parasite reservoir [25]. However, sufficiently sensitive, field-adaptable, high-throughput detection kits are needed to reduce analysis times and allow timely planning of interventions.

## Conclusion

Dry season parasite and serological surveys are useful tools for identifying malaria foci as well as for describing short and medium term trends in malaria transmission. Both tools are able to detect heterogeneity in transmission across the country though their results are weakly correlated. Field-adapted serological tests would be extremely useful as a complement for mass screening and treatment programs.
